# Induced Pluripotent Stem Cells Generated from Human Adipose-Derived Stem Cells Using a Non-Viral Polycistronic Plasmid in Feeder-Free Conditions

**DOI:** 10.1371/journal.pone.0048161

**Published:** 2012-10-26

**Authors:** Xinjian Qu, Tianqing Liu, Kedong Song, Xiangqin Li, Dan Ge

**Affiliations:** Dalian R&D Center for Stem Cell and Tissue Engineering, Dalian University of Technology, Liaoning, Dalian, China; University of Milan, Italy

## Abstract

Induced pluripotent stem cells (iPSCs) can be generated from somatic cells by ectopic expression of defined transcription factors (TFs). However, the optimal cell type and the easy reprogramming approaches that minimize genetic aberrations of parent cells must be considered before generating the iPSCs. This paper reports a method to generate iPSCs from adult human adipose-derived stem cells (hADSCs) without the use of a feeder layer, by ectopic expression of the defined transcription factors *OCT4*, *SOX2*, *KLF4* and *C-MYC* using a polycistronic plasmid. The results, based on the expression of pluripotent marker, demonstrated that the iPSCs have the characteristics similar to those of embryonic stem cells (ESCs). The iPSCs differentiated into three embryonic germ layers both *in vitro* by embryoid body generation and *in vivo* by teratoma formation after being injected into immunodeficient mice. More importantly, the plasmid DNA does not integrate into the genome of human iPSCs as revealed by Southern blotting experiments. Karyotypic analysis also demonstrated that the reprogramming of hADSCs by the defined factors did not induce chromosomal abnormalities. Therefore, this technology provides a platform for studying the biology of iPSCs without viral vectors, and can hopefully overcome immune rejection and ethical concerns, which are the two important barriers of ESC applications.

## Introduction

Reprogramming differentiated human cells to induced pluripotent stem cells (iPSCs) has attracted much attention because the iPSCs facilitate the production of patient-specific stem cells, that can hopefully overcome immune rejection and ethical concerns, the two important obstacles in subsequent clinical applications [1]–[3]. But, from generation of iPSCs generation to making them amenable to clinically applications, several challenges remain to be addressed. The main challenges include, effective methods to deliver defined factors in reprogramming process, availability of the cell types for easy introduction of factors without acquired DNA damages, and optimal culture conditions for deriving iPSCs [4]. Previously devised strategies for production of iPSCs have so far been mainly through retroviral vectors and constitutive lentiviral systems. These viral systems, however, have been criticized for their permanent integration into the genome. Therefore it is necessary to pursue non-integration approaches.

With fast progress in this field, different reprogramming strategies have been developed, including the use of non-integration adenoviruses, reprogramming with a polycistronic cassette containing all four factors, excisable transposons, and virus-free plasmids [5]–[11]. Initially, a non-viral vector approach to generate iPSCs was developed to improve efficiency, which required two individual plasmids to deliver transcription factors (TFs) [7], [12], and the subsequent removal of the viral genome by *loxp/Cre* or *Piggybac transposons* [4]–[7]. Furthermore, a single vector encoding a stem-cell cassette was created,which expresses the four TFs from a single multicistronic transcript. Although these methods solved some of the limitations posed by the presence of multiple proviruses in the genome of iPSCs, they either involved insertional mutagenesis or complicated procedures. So far, single polycistronic lentiviral vector reprogramming of murine and human somatic cells has been reported [13], but there is no report about polycistronic virus-free plasmid transduced human somatic cells.

Eversince the first iPSCs were generated, a multitude cell types like stomach cells, neural progenitor cells have been successful reprogrammed [14]–[16]. During the process, investigators endeavored to employ genetic labeling or other techniques to confirm the donor cells so as to rule out the possible contamination of resident feeder cells [17], [18]. Additionally, for a given optimal cell type, several factors must be considered that the cell types should be easily attainable, not contain genetic aberrations, and easy to be reprogrammed with transient approaches [19]. Therefore, it is essential to improve the methodologies for iPSC generation and to avoid xenobiotic contamination for safety and efficacy [4]–[6], [20], [21].

Adipose derived stem cell (ADSC) can be regarded as an excellent candidate for cell therapy due to its easy accessibility, isolation, and being expandable to clinical scales in a comparatively short period of time [22]. In addition, ADSCs can be bio-preserved for point-of-care delivery with minimal loss of ‘stemness’. Human trials based on the ADSCs thus far have shown no adverse reactions to allogeneic versus autologous cellular transplants, enabling the creation of an inventory of ideal donors [17], [23], [24].

Two independent groups [7], [17] have reported the generation of iPSCs by transgenic manipulation of mouse and human adipose derived stem cells. In their studies, the same “Yamanaka” genes were able to establish the iPSCs under feeder-free conditions, with iPSCs’ properties almost indistinguishable from those of ESC [10]. However, a critical limit of these reports was the use of retroviral/lentivirus-mediated systems, which increases the risk of tumor formation due to genomic insertion.

From the perspectives of safety and efficiency, a polycistronic plasmid method was applied to generation of iPSCs from ADSCs. Taking pIRES2-EGFP plasmid as a basic backbone, we constructed a polycistronic vector using self-cleaving 2A sequences and the constitutively active encephalomyocarditis virus (CMV) promoter. Four “Yamanaka” genes were inserted into the vector in oskm-2A order. Fortunately, iPSCs could be readily derived from hADSCs in feeder-free environment with ectopic expression of the “Yamanaka” four factors. The iPSCs clones expressed major ES cell markers and contributed to forming teratomas that differentiated into all three germ layers, indicating that ADSCs have the potential in regenerative therapeutics especially in feeder-free iPSC generation and maintenance.

## Results

### A Polycistronic Plasmid Vector for the Generation of iPSC Lines

**Figure 1:**
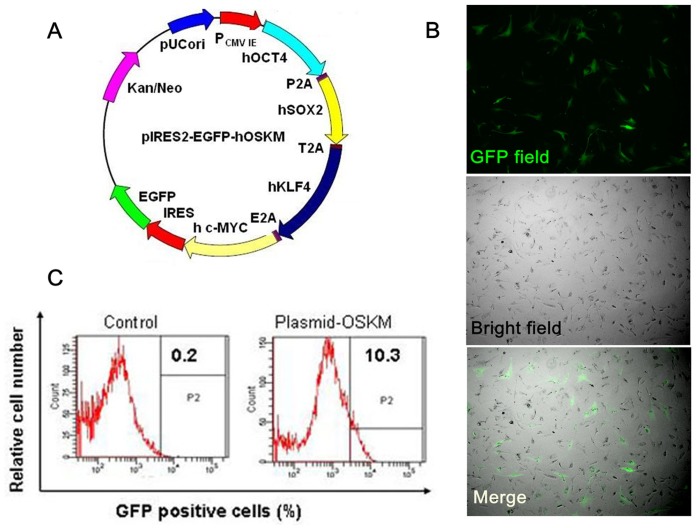
Generation of a polycistronic expression vector for iPSC generation. (**A**) Schematic representation of plasmid vector and 2A-linked fusion gene (*OSKM*). Four defined TFs (*OCT3/4*, *SOX2*, *KLF4*, and *c-MYC*) were fused in-frame via 2A sequences and co-expressed as a single ORF. (**B**) Comparative representation of GFP expression in hADSCs 48 h after transfection of OSKM-plasmid. GFP positive fluorescence (left panel, 40×magnification), phase contrast (middle panel, 40×magnification) and merge (right panel, 40×magnification) micrographs are shown. (**C**) Representation of flow cytometry analysis of GFP-expressing hADSCs 6 days after transfection.

Our goal was to generate polycistronic plasmid vectors that could express multiple reprogramming genes from a single promoter using 2A peptides. The self-cleaving 2A peptides are very small in size and can efficiently cleave polycistrons at specific sites. To construct a polycistronic plasmid vector, cDNAs coding for the human *OCT4, Sox2*, *Klf4*, and *c-Myc* TF genes were joined with self-cleaving 2A sequence as a fusion gene (*OSKM*) within a single open reading frame (ORF). Then, this ORF was cloned upstream of an internal ribosome entry site (IRES) in the pIRES2-EGFP plasmid and driven by a common CMV promoter (P_CMV IE_) (Fig. 1A). The GFP gene was promoted by IRES, because IRES permits both the upstream *OSKM* gene and downstream GFP gene to be translated from a single mRNA. The vector backbone also contains an SV40 origin for replication in mammalian cells expressing the SV40T antigen. A neomycin-resistance cassette (Neo^r^) of plasmid vector allows transfected eukaryotic cells to be selected using G418.

Therefore, derived from the pIRES2-EGFP plasmid vector can be transfected into somatic cells without the need for viral packaging and can be subsequently removed from cells by culturing in the absence of drug selection. More importantly, the plasmid vector cannot replicate in hADSCs because hADSCs cannot express the SV40T antigen. All of these properties made the pIRES2-EGFP plasmid vector more suitable for introducing defined factors into human ADSCs.

To test whether the polycistronic plasmid was to be expressed in somatic cells, hADSCs were transfected with *OSKM*-plasmid by using Calcium-phosphate-mediated Transfection Method. The GFP marker was clearly visible by fluorescence microscopy 48 hours post-transfection (Fig. 1B). About 50% of hADSCs were transiently transfected as estimated by GFP expression. A second round of transfection was then performed on day 4, so that nearly all of cells received at least one transient plasmid transfection. Two days later, the cells were passaged and propagated subsequently. In the next 4 days, FACs analysis demonstrated one-tenth GFP positive cells indicating that these cells had been transfected by the polycistronic plasmid-OSKM (Fig. 1C).

### iPSCs Generated by Using OSKM-plasmid

**Figure 2:**
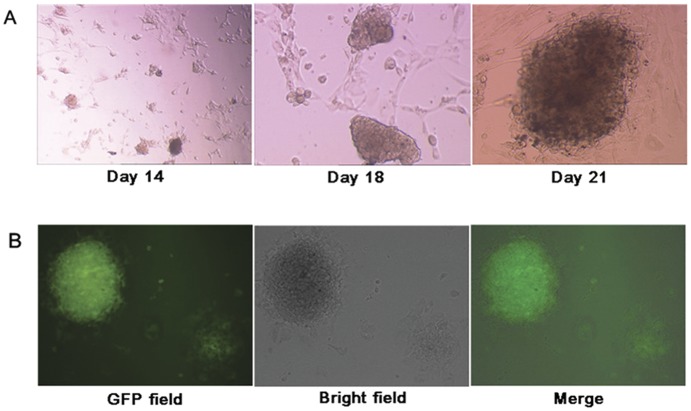
Generation of iPSC colonies from hADSCs with the *OSKM* fusion gene introduced. (**A**) Morphological changes in the OSKM-transfected ADSCs under bright-field microscopy during the reprogramming process at 14 (left panel, 40×magnification), 18 (middle panel, 100×magnification) and 21 days (right panel, 100×magnification) after the second transfection. (**B**) GFP positive colonies were observed by day 18 after the second transfection. The morphology of the ADSCs derived iPSCs in GFP positive fluorescence (left panel, 100×magnification) and in phase contrast (middle panel, 100×magnification), and merged image (right panel, 100×magnification).

As has been reported earlier [10], human iPSC colonies can be readily identified based on the expression of pluripotent markers and characters of ES cell-like morphology. In all of our experiments, the control cells transfected with empty pIRES2-EGFP plasmid did not change their morphology and continued to grow as a monolayer. In contrast, the cells transfected with the OSKM-plasmid not only exhibited GFP expression, but also formed more aggregates with upheaval-shaped morphology while propagating after two weeks after transfection (Fig. 2A and B), which were distinct from hADSCs. These gathered cells adopted a tightly packed morphology and formed a number of undifferentiated colonies at 3–4 weeks after transfection. The obvious features of the colonies were their refractive edges morphology as well as three-dimensional growth highly reminiscent of those of human ESC colonies.

**Figure 3:**
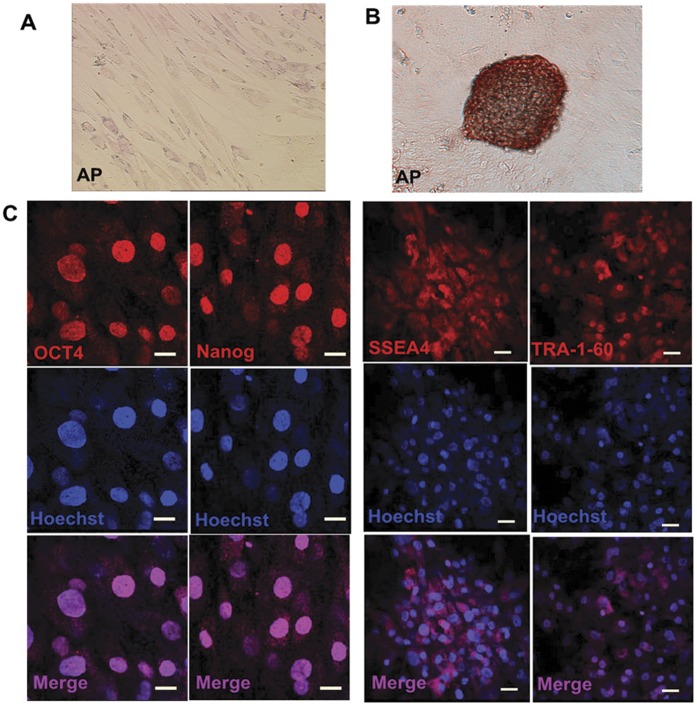
Generated iPSCs expressed pluripotent markers. (**A**) Alkaline phosphatase staining of the ADSCs indicated the cells expressed AP activity. The ADSCs without the *OSKM* fusion gene were used as control cells. (**B**) The AP straining of early formed clones by *OSKM* induced iPSC showed tight, upheaval-shaped morphology 14 days after transgenesis. (**C**) Human ES cell-specific surface antigen staining of iPSCs. Immunofluorescent staining results demonstrated that the iPSCs were positive for OCT4, NANOG, SSEA-4, and TRA-1-60 (red), Hoechst (blue), and merged image.

To test whether pluripotency was induced, three colonies (iPS1, iPS2 and iPS3) were isolated and expanded for further analysis. Figure 3 shows that hADSCs express alkaline phosphatase (AP) activity weakly (Fig. 3A), whereas the generated colonies not only exhibited AP activity strongly (Fig. 3B), but also showed expression of ES cell-associated pluripotency markers, including TRA-1-60, OCT4, Nanog and SSEA4 in 28 days (Fig. 3C). Thus, the ES-like morphology, in combination with ESC antigen staining suggests that these colonies are likely to have been reprogrammed to an ES-like state.

**Figure 4:**
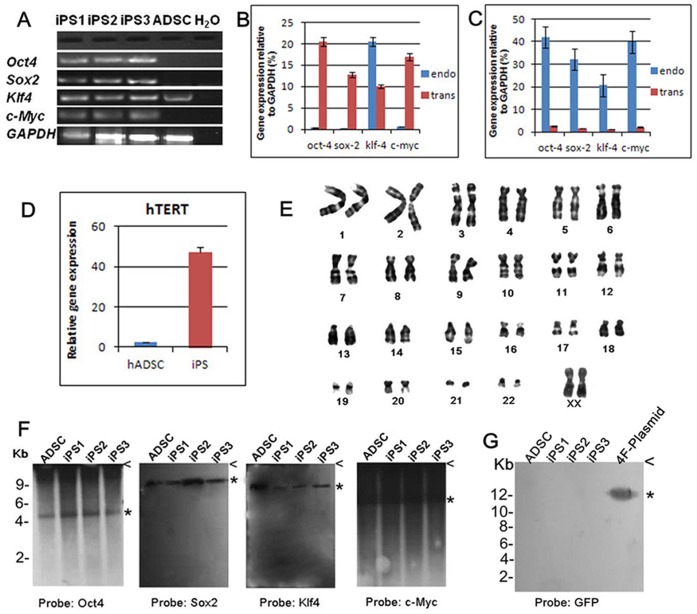
Generated iPSCs expressed pluripotent marker genes and did not contain plasmid victor integration. (**A**) Molecular characterization of iPSC lines. RT-PCR analyses by using primers specific to the endogenous of human cells demonstrated all defined 4 TF genes expressed in the three iPSC lines, while hADSCs expressed Klf4 only. (**B**) q-PCR analyzed the expression levels of transgenic and endogenous defined factors in passage 2 iPSCs, relative to GAPDH (100%) expression. (**C**) q-PCR analyzed the expression levels of the transgenic and endogenous TFs in passage 5 iPSCs, relative to GAPDH (100%) expression. (**D**) q-PCR analyzed the expression of pluripotency-related genes hTERT in generated iPSCs relative to hADSCs expression. (**E**) Karyotype of generated iPSCs was normal 46 XX. (**F, G**) Southern blot analysis test plasmid integration in iPSC genome. ADSCs (as control) and iPSCs DNA was digested with BamHI. Hybridization of the same molecular weight fragment using all four probes indicated the presence of no plasmid sequence insertion (**F**). No clones carried the plasmid DNA sequence indicated plasmid probe detection (**G**).

To assess whether or not the endogenous *OSKM* genes were reactivated in hADSC derived iPSC colonies, we analyzed the *OSKM* with polymerase chain reaction with reverse transcription (RT-PCR) by using primers specific for these four genes. The results demonstrated all defined 4 TF genes were expressed in all three iPSC lines, while hADSCs expressed Klf4 only (Fig. 4A).

To further assess the expression level of endogenous and exogenous *OSKM* genes in hADSCs-derived iPSCs, we selected iPS1 as a sample in passages 2 and 5 by quantitative PCR (q-PCR) analysis. In passage 2, Oct4, Sox2 and c-Myc transgenes were expressed at high level in comparison to the endogenous genes. In contrast, the level of endogenous Klf4 gene expression was higher than that of the transgene (Fig. 4B). In cells at passage 5, expression levels of all the four endogenous genes were higher than those of the transgenes (Fig. 4C). Quantitative-PCR experiments examining the expression of pluripotency-related gene hTERT showed an increased expression in generated iPSCs compared to hADSCs (Fig. 4D). In addition, karyotypic analysis revealed that chromosomal abnormalities did not arise in passage 5 as a result of reprogramming (Fig. 4E). To exclude the possibility of the plasmid vector integration into the genomes of the iPSC clones, genomic DNA was extracted and subjected to Southern blot analysis. Both four factor gene probes and GFP probe were applied in the hybridization experiments, and results demonstrated that plasmid sequence was not inserted into iPSCs genomes (Fig. 4F and G).

In general, reprogramming efficiency was calculated as the percentage of the number of iPSC colonies counted in the seeded transfected hADSCs. But in this experiment, neither was drug selection used nor was exogenous feeder layer cells adopted. iPSC colonies were thus identified based on ES cell-like morphology and GFP expression at the beginning. We consistently observed ≈40 GFP positive, ES cell-like colonies out of 60,000 cells on days 21. Then, the colonies were propagated and passaged twice a week for at least 2 months. They maintained ES cell-like morphology while the expression of GFP decreased gradually because the plasmid vector was gradually lost from proliferating cells in the absence of drug selection. Therefore in this study, we combined the ES cell-like morphology and pluripotent marker analysis (including TRA-1-60, OCT4, Nanog and SSEA4) to determine the reprogramming efficiency in the subsequent stages. Later, all colonies were successfully expanded and cultured in feeder-free condition for an extended time. Using Immunofluorescent staining of pluripotent markers, quantitative PCR and karyotype analysis shows that these iPSC lines expressed typical hES cell markers and sustained normal karyotypes. As a result, the reprogramming efficiency was about 0.006±0.0005%.

### Embryoid Body and Teratoma Formation

**Figure 5:**
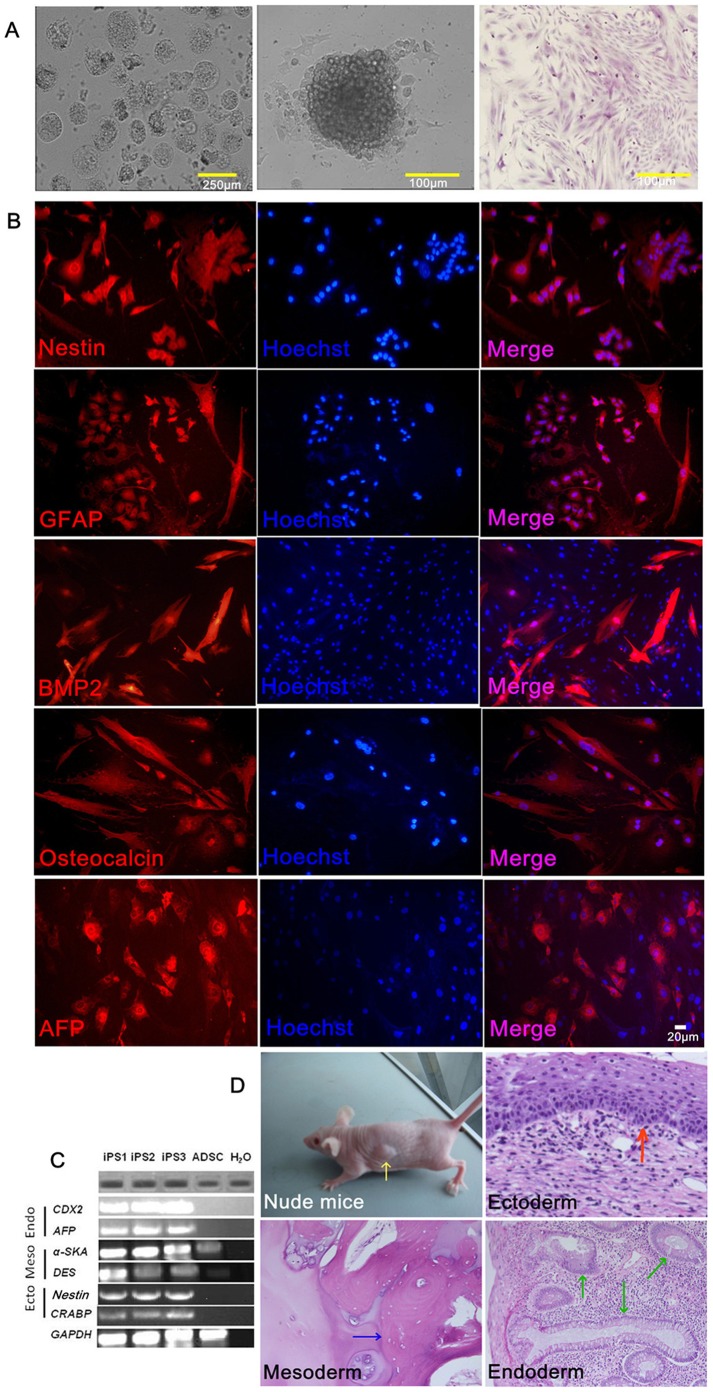
ADSCs-derived-iPSCs were pluripotent and differentiated *in vitro* and *in vivo*. (**A**) Phase-contrast images of EBs generated from iPSC clones in suspension culture. EB was formed in suspension culture for 3 days (left, 40×magnification). EBs were allowed to differentiate further in adherent culture for 2 days (middle, 100×magnification). H&E staining of different cell types during EB differentiation after adherent culture continued for 5 days (right,40×magnification). (**B**) immunofluorescence staining of differentiated cells derived from iPSCs with antibodies specific for lineage-specific markers: Nestin and GFAP (ectoderm marker), BMP2 and Osteocalcin (mesoderm marker) and AFP (endoderm marker)(100×magnification). (**C**) RT-PCR analysis of differentiation markers from iPSC-derived EB showed differentiated three germ layers, including Nestin and CRABP2 (ectoderm), α-SKA and DES (mesoderm), CDX2 and AFP (endoderm). (**D**) H&E staining of histological sections through teratomas formed by iPSCs. They formed all three embryonic germ layers including gland (endoderm), bone (mesoderm), and stratified squamous epithelial (ectoderm) tissues. Teratoma (40×magnification).

To investigate the pluripotency of the generated iPSCs, embryoid body (EB) experiments were performed. By using standard protocols used for ESC differentiation, these iPSC clones formed spherical EB structures after 8 days in suspension culture with differentiation medium. After growing in suspension for 8 days, the EBs were replated in adherent conditions and driven to differentiate under various conditions (Fig. 5A). Immunocytochemical analysis of EBs after 10 days of culture showed distinctive morphologies under standard EB or directed differentiation conditions, with markers indicative of all three germ layers- ectoderm (Nestin, GFAP), mesoderm (BMP2, Osteocalcin), and endoderm (AFP) (Fig. 5B). RT-PCR experiments to analyze the expression of genes specific for ectoderm, endoderm, and mesoderm, respectively, revealed the cellular ability to up-regulate different lineage marker genes (Fig. 5C). Nestin and CRABP2, markers of epidermis and the ectodermal lineage, were also induced under certain conditions (Fig. 5C). Mesodermal differentiation was revealed by expression of α-SKA and DES, whereas endodermal differentiation was highlighted by CDX2 and AFP expression (Fig. 5C). In contrast, α-SKA was expressed at lower levels in hADSCs under various induced conditions (Fig. 5C) [15].

Human ES cells form teratomas when injected into immunocompromised mice. This has become a standard assay for pluripotency. To test the development potential of ADSC-iPSCs, we then transplanted the iPSCs subcutaneously into nude mice and found that all the iPSCs generated teratomas 7 weeks after transplantation. Hematoxylin and eosin (H&E) staining indicated that the iPSCs could differentiate into a variety of cell types from all three germ layers, including glands, bone, and stratified squamous epithelial cells, thus demonstrating the pluripotency of ADSC-iPSCs (Fig. 5D).

## Discussion

An important justification for this paper is that it is the first study to report the establishment of human ADSC derived iPSCs using a polycistronic plasmid method. Compared to previously reported plasmid strategies [8]–[10], reprogramming by a polycistronic plasmid vector offers several advantages. First, it is the use of CMV constitutively active promoter, which is more susceptible to silencing exogenous genes when somatic cells are reprogrammed into pluripotent state [25]. Second, the selection of pIRES2-EGFP as basic plasmid backbone is another character because it couldn’t self-replicate in mammalian cells, except in those cells expressing SV40T-antigen. Finally, our study included technically simple procedures, demonstrates gradual loss of plasmid in cell divisions progress, used a single polycistronic plasmid carrying all four Yamanaka factor genes.

While this manuscript was in review, an alternative approaches was described under which oriP/EBNA1 was used to excise integrated transgenes from human iPS cells [26]. Comparatively, there are some similar characters and mechanisms in delivery gene system between oriP/EBNA1 vector and pIRES2-EGFP vector. Both the vectors are extrachromosomal replication plasmid and involved in some important elements such as P_CMV_, SV40pA, IRES and oriP. Moreover, we got the similar result that was the absence of the vector sequences in the genomic of the generated iPSC clones. But, a significant difference was that oriP/EBNA1 vector could be replicated once per cell cycle in mammalian cells while pIRES2-EGFP vector couldn’t self-replicate except those mammalian cells expressing SV40T antigen.

Reprogramming efficiency is one of the most important issues in iPSC studies [20], [26], [27]. In our experiments, we improved the reprogramming efficiency by double transfections. In this way, the efficiency of our iPSC generation (0.006%–0.01%) is lower than that of the retroviral or the lentiviral infection approaches (0.1–1%) [25], but relatively higher than the two other reported efficiencies using plasmid vectors protocols (0.001–0.01%) [4]. More importantly, since our work was done under feeder free conditions, the effectiveness and the efficiency of generating iPSCs was partly ascribed to the selected cell type.

**Figure 6:**
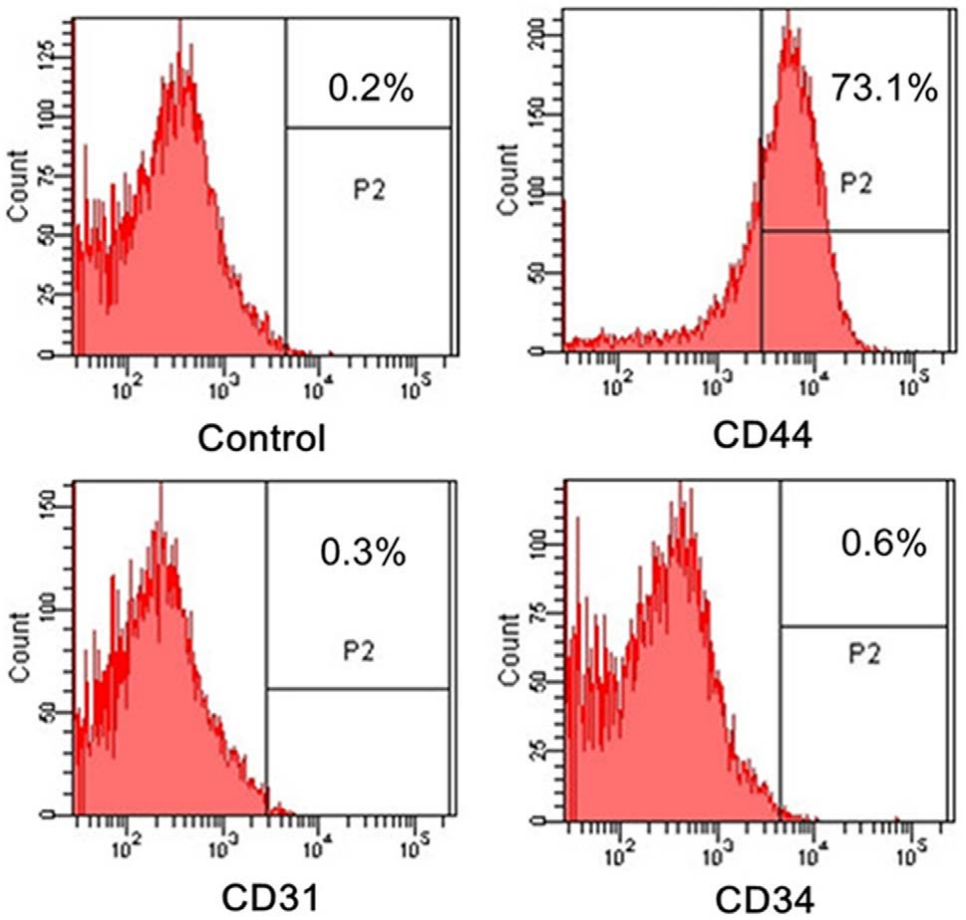
Representative histograms of FACS analysis showing hADSCs expressed mesenchymal stem cell marker CD44, but not any of the endothelial cells marker CD31 and hematopoietic cells marker CD34.

hADSCs have some unique characters that other types of somatic cells may not possess. They strongly express AP and Klf4. FACS analysis also showed hADSCs expressed mesenchymal stem cell marker CD44, but not any of the endothelial cells marker CD31 and hematopoietic cells marker CD34 (Fig. 6). It has been reported that chromatin remodeling is a restrictive element in the conversion from a somatic to a pluripotent epigenetic state [15]. Meanwhile, Klf4 plays a major role in chromatin remodeling, and recent evidence demonstrates that Klf4 is an important mediator of the undifferentiated ES cell state. Therefore, compared to terminally differentiated cells, hADSCs have an epigenomic regulatory pattern that is closer to pluripotent cells. Hence hADSCs may present fewer barriers for reprogramming. Previous studies reported that hADSCs not only expressed pluripotency genes OCT4, Klf4, but also Klf2, Esrrb and c-MYC, while our results here did not show Oct4 and c-MYC activity at the mRNA level. The underlying mechanism may be due to donor age, differentiation stage, lipoaspiration region, or various culture conditions [28].

Recently, feeder-free layers culturing iPSC derived from ADSCs attracted wide attention [1], [4], [10]. ADSCs intrinsically secrete various pluripotency-sustaining cytokines, such as vascular endothelial growth factor (VEGF),TGFβ, fibronectin-1 and vitronectin [16]. More importantly, ADSCs also express higher levels of bFGF and LIF that play critical roles in supporting proliferation and self-renewal of ES/iPS cells [10]. This might explain the possible mechanism of reprogramming in the feeder-free condition. So far, only a few types of cells have been induced into pluripotency in the complete absence of cocultured feeder cells [29].

Here, we demonstrated that our protocols not only were capable of reliably reprogramming adult ADSCs into iPSCs without exogenous feeder layers, but provided no viral delivery approaches and technically simple procedure advantages. Besides, our results offer more cognitive schema of ADSCs that facilitates its application and further studies [30].

## Materials and Methods

### Polycistronic Plasmid Vector Construction

*OCT4, Sox2*, *Klf4*, and *c-Myc* were amplified by polymerase chain reaction (PCR) using Prime STAR HS DNA polymerase (*TaKaRa* ***)***. Open reading frames of individual genes were fused to P2A, T2A, and E2A sequences (*Oct4- P2A -Sox2- T2A -Klf4- E2A-cMyc*) (19,20), as a single open reading frame (ORF) designated as *OSKM*. Respective 2A sequences: P2A, GCC ACG AAG CAA GCA GGA GAT GTT GAA GAA AAC CCC GGG CCT; T2A, GAG GGC AGA GGA AGT CTT CTA ACA TGC GGT GAC GTG GAG GAG AAT CCC GGC CCT; and E2A, ACG TGT ACT AAT TAT GCT CTC TTG AAA TTG GCT GGA GAT GTT GAG AGC AAC CCA GGT CCC. The *OSKM* fusion genes were cloned into the pIRES2-EGFP to form a polycistronic expression vector. The resulting plasmid was designated as pIRES2-EGFP-OSKM. The plasmid vector backbone also contains a neomycin-resistance cassette (Neo^r^), consisting of the neomycin/kanamycin resistance genes that can be used to select stably transfected eukaryotic cells using G418. Plasmid vectors were transfected using Calcium-phosphate-mediated Transfection Method following the manufacturers’ directions. One day after transfection, the medium was replaced with fresh culture medium. Medium was changed every 2 days.

### Cell Isolation

The ADSCs were isolated from subcutaneous fat of a 30-year-old female by digestion at 37°C for 20 minutes with 0.25% trypsin and 0.1% type I collagenase in Hank’s buffered salt solution (v/v 1∶1), detail of the work conformed to the provisions of the Declaration of Helsinki (as revised in Edinburgh 2000). After sequential filtration through 250- and 100-µm nylon filters and centrifugation for 10 min at 2000 rpm, the adipocytes were washed three times, and seeded at a density of 1.25×10^5^ cells/well in gelatinized (0.1%) 6-well plates, where the DMEM (Invitrogen) with 10% FBS (GIBCO) and penicillin-streptomycin was added. The cells were passaged at least twice before to remove non-adherent cells.

### Transfection and Cell Culture

Human ADSCs were isolated and then enriched by serial plate passages, before the transgenes encoding *OCT4*, *SOX2*, *KLF4* and *c-MYC* were introduced at passage 3. Briefly, 24 hours before transfection, exponentially growing cells were harvested by trypsin and replated at a density of 1.5×10^5^ in each 4 wells of 6-well plates in the appropriate complete medium. The calcium phosphate-DNA coprecipiate was prepared as follows: combine 100 µl of 2.5 M CaCl_2_ with 25 µg of plasmid DNA in a sterile 5-ml plastic tube, mix this complex with an equal volume of 2×HEPES-buffered saline at room temperature. Immediately the calcium phosphate-DNA suspension was transferred into the medium above the cell monolayer. After 4 hours of incubation, the medium and DNA precipitate were remove by aspiration and warmed complete growth medium was added. These cells were transfected twice over 4 days with the same volume of plasmid DNA, and cultured in standard ADSCs medium, then passaged in an ES cell-supportive medium without feeder cell layers. GFP positive cells expressing transgenes were detected after 48 h transduction. We optimized the reprogramming protocols with the following modifications. (i) The cells were seeded in ADSCs medium that contain fresh DMEM-F12 medium and the MEFs culture medium (v/v, 1∶1). (ii) the transfected hADSCs were cultured on G418-independent medium without feeder layer cells. This medium was supplemented with L-glutamine, nonessential amino acids, penicillin-streptomycin, knockout serum replacement, and 10 ng/ml basic FGF.

### RT-PCR and qPCR

Total RNA was isolated using RNeasy kit followed by cDNA synthesis using Superscript Reverse Transcriptase and Oligo (dT) 12–18 primers. PCR was performed with Start Taq DNA polymerase. Quantitative PCR was performed using QuantiFast SYBR Green PCR Kit and analyzed with RotorGen. All reagents were from *TaKaRa*. The following primers were used [14]:

Endogenous:

OCT4 (NM_002701): 5′-ACTCCTGCTTCGCCCTCA-3′, 5′-TCGGATTTCGCCTTCTCG-3′.

SOX2 (NM_003106): 5′-TCGCAGCCGCTTAGCCTCGT-3′,5′-AACAGCCCGGACCGCGTCAA-3′.

Klf4 (NM_004235): 5′-GACTCACCAAGCACCATC-3′, 5′-CAGCCAGAAAGCACTACAA-3′.

c-Myc (NM_002467): 5′-CCTCATCTTCTTGTTCCTCCT-3′, 5′-ACAGCGTCTGCT CCACCT-3′.

Transgenous:

OCT4: 5′- GAAGGATGTGGTCCGAG-3′, 5′- CTTCGTGGCTCAGTTTG -3′.

SOX2: 5′-GCTCCATGGGTTCGGTCAA-3′, 5′- ATGGTTAGAAGACTTCCTCT -3′.

Klf4: 5′- ATCCCGGCCCGATGGCTGTCAGC -3′, 5′- ATAATTAGTACACTGGTAGC -3′.

c-Myc: 5′- TGTTGAGAGCAACCCA -3′, 5′- TCTCCCCGAAGGGAGAA -3′.

CRABP2 (NM_001878): 5′- AGTGAAGCAGGGCGGTGA-3′, 5′- AGGAGCAGACTGTGGATGG-3′.

DES (NM_001927 ): 5′- CGGAAGTTGAGGGCAGAGT-3′, 5′- CCTGAAGGGCACTAACGATT-3′.

CDX2 (NM_19920): 5′- TCCGCATCCACTCGCACA-3′, 5′- CGCCGCAGAACTTCGTCA-3′.

AFP (NM_001134): 5′- TAGCGAGCAGCCCAAAGA-3′, 5′- AAAGCCCACTCCAGCATC-3′.

Nestin (NM_006617): 5′- ACCCTTGCCTGCTACCCT-3′, 5′- CCCTCTATGGCTGTTTCTTTC-3′.

α-SKA (NM_019183): 5′- ACCAGGGTGTCATGG-3′, 5′- GTGAGCAGGGTCGGG-3′.

GAPDH (NM_017008): 5′- ATTGTCAGCAATGCATCCTG-3′, 5′- GTAGGCCATGAGGTCCACCA-3′.

### Southern Blotting

Ten micrograms of genomic DNA was isolated and digested with BamHI from each of the three iPSC subclones derived from human ADSCs (iPS1, iPS2 and iPS3). As negative controls, hADSCs genomic DNA was treated as introduced above. After digestion, DNA was separated on a 0.7% agarose gel in TAE buffer pH 8.4 then capillary-transferred overnight onto a positively charged BrightStar-Plus nylon membrane (Ambion) in alkaline solution (0.4 M NaOH and 2 M NaCl). Hybridization was performed using synthesized biotin-labeled probes of OCT4, Sox2, Klf4, c-Myc and GFP then detected with the Biostar-BioDetected Kit (Ambion). The following primers were used for cloning DNA fragments which as DNA templates in DNA probes synthesis proceed.

OCT4 (NM_002701): 5′- GGATTTCGCCTTCTCGC -3′, 5′-ACAGTGCAGTGAAGTGAG-3′.

SOX2 (NM_003106): 5′- TACAACATGATGGAGACGGA -3′, 5′- ATGGCCGTGCCGGGCAC-3′.

Klf4 (NM_004235): 5′-AGACACTGCGTCAAGCAGGTG-3′, 5′- ACAGCCGTCCCAGTCACAGT-3′.

c-Myc (NM_002467): 5′-ATGACCTCGACTACGACTC-3′, 5′- TCTTGGCAGCAGGATAGTCCTTC-3′.

GFP: 5′-ACGTAAACGGCCACAAGTTCAGC-3′, 5′-TAGTGGTTGTCGGGCAGCAGCAC-3′.

### Karyotype Analysis

The cells were cultured for 24 h in a CO_2_ incubator, and treated with colcemid (0.1 µg/ml, final concentration) for four hours before being harvest. The cells were trypsinized and transferred into 15 ml tubes. They were then centrifuged for 10 min at 1000 rpm and the supernatant was removed and resuspended with 10 ml KCl solution (75 mM). The cell mixtures were incubated for 30 min in 37°C water bath and then fixed by adding 4 ml fixative solution (methanol/acetic acid 3∶1). The fixed cells were washed twice with 10 ml of fixative solution before being applied onto chilled slides. The slides with chromosomes were dried and treated with 0.25% typsin for 5 min and stained with Giemas (1∶10) for 10 min.

### In vitro Differentiation

hADSCs derived iPSCs cultured on Matrigel were treated with collagenase type IV (Invitrogen) and transferred to ultra-low attachment plate (Corning Life Sciences) in suspension culture for 8 days with DMEM/F12 (1∶1) containing 20% FBS (HyClone), 4.5 g/L L-glutamine, 1% nonessential amino acids, 0.1 mM β-mercaptoethanol, 50 U/ml penicillin, and 50 µg/ml streptomycin. EBs was then seeded in 0.25% gelatin-coated tissue-culture dishes in different culture medium for differentiation.

For ectoderm differentiation, EBs was cultured in N2B27 medium containing bFGF (20 ng/ml) and EGF (20 ng/ml). After 7 days, ectodermal markers were detected.

For mesoderm differentiation, cells were cultured in LG-DMEM medium supplemented with 10% FBS, 300 µg/ml L-glutamine,50 µg/ml vitamin C,10 mM β-phosphoglycerol, 10 nM vitamin D for mesoderm differentiation. The media were changed every 3 days. Two weeks later, morphological changes were visible.

For endoderm differentiation, cells were culture as a monolayer in endoderm differentiation medium (DMEM, B27 (-RA), and 100 ng/ml activin-a) for 7 days. Then the medium was switched to growth medium (DMEM, 10%FBS) and the continued differentiation lasted for 7 days.

The differentiation of hADSC-iPSCs into three germ layers was then detected with appropriate markers by immunofluorescence and RT-PCR.

### Teratoma Formation

Human iPSC lines were collected by collagenase type IV treatment and suspended in DMEM containing 10% FBS. Then 100 µl (5×10^5^ cells) suspensions were injected subcutaneously into both dorsal flanks of nude mice anaesthetized with isoflurane. Palpable tumors were observed typically 6 weeks after injection. The teratomas were dissected 9 weeks after injection, fixed overnight in 10% buffered formalin phosphate and embedded in paraffin. Sections were stained with haematoxylin and eosin. The experiments involving animals conform to national guidelines for animal usage in research.

### Immunostaining and Flow Cytometry

Cells were grown on gelatin-coated 6-well plates. Samples were washed with PBS, fixed in 4%PFA/PBS for 10 min at 25°C, permeabilized with 0.3% Triton X-100 in PBS for 10 min at 25°C, blocked in 5% goat serum for 1 h. Then the following primary antibodies were added overnight at 4°C the primary antibodies for OCT4 (1∶50, #886906, Abcam), Nanog (1∶100, #885871, Abcam), TRA-1-60 (1∶100, #sc-21705, Santa Cruz), SSEA-4 (1∶100, #sc-21704, Santa Cruz), AFP (1∶100, #sc-51506, Santa Cruz), GFAP (1∶100, #sc-9065, Santa Cruz), Nestin (1∶100, #250764,Abbiotec), BMP-2 (1∶100, KG22178-2, KeyGen), Osteocalcin (1∶100, KG22596-2, KeyGen). The cells were washed with PBS and then incubated with secondary antibody (PE-conjugated) diluted 1∶100 for 45 min at 37°C. Secondary antibodies: Goat anti-mouse lgM-PE (1∶100, #sc-3768, Santa Cruz) or Goat anti-rabbit lgG-PE (1∶100, #sc-3739, Santa Cruz). Fluorescence imaging was performed using Olympus AS-70 microscope, Olympus Confocal Scanning Laser Microscope (FV1000) and Image-Pro Plus Software.

Alkaline phosphatase staining was performed by using alkaline phosphatase detection kit (Millipore).

For flow cytometric analysis, cells were detached and fixed in methanol, washed with PBS twice to detect GFP positive cells. For surface marker analyses, human cells were labeled with fluorescence-conjugated anti-human antibodies, PE-CD44 (BD Biosciences), PE-CD31 (BD Biosciences) and PE-CD34 (BD Biosciences), according to the manufacturer’s instructions. Flow cytometric data were analyzed using CellQuest Software (Becton Dickinson). For each sample, at least three independent experiments were performed.

### Statistical Analysis

All the data are expressed as the mean ± standard error unless otherwise stated. Statistical analysis was performed on the data using Microsoft Excel. For comparisons of discrete data sets, unpaired Student’s *t*-tests were used. Significance levels or *P*-values were stated in figure legends.
